# Comparative Genomics of the Conjugation Region of F-like Plasmids: Five Shades of F

**DOI:** 10.3389/fmolb.2016.00071

**Published:** 2016-11-10

**Authors:** Raul Fernandez-Lopez, Maria de Toro, Gabriel Moncalian, M. Pilar Garcillan-Barcia, Fernando de la Cruz

**Affiliations:** ^1^Instituto de Biomedicina y Biotecnologia de CantabriaSantander, Spain; ^2^Centro de Investigacion Biomedica de la RiojaLogroño, Spain

**Keywords:** plasmids, plasmid conjugation, IncF incompatibility group, plasmid evolution, antibiotic resistance

## Abstract

The F plasmid is the foremost representative of a large group of conjugative plasmids, prevalent in *Escherichia coli*, and widely distributed among the Enterobacteriaceae. These plasmids are of clinical relevance, given their frequent association with virulence determinants, colicins, and antibiotic resistance genes. Originally defined by their sensitivity to certain male-specific phages, IncF plasmids share a conserved conjugative system and regulatory circuits. In order to determine whether the genetic architecture and regulation circuits are preserved among these plasmids, we analyzed the natural diversity of F-like plasmids. Using the relaxase as a phylogenetic marker, we identified 256 plasmids belonging to the IncF/ MOB_F12_group, present as complete DNA sequences in the NCBI database. By comparative genomics, we identified five major groups of F-like plasmids. Each shows a particular operon structure and alternate regulatory systems. Results show that the IncF/MOB_F12_ conjugation gene cluster conforms a diverse and ancient group, which evolved alternative regulatory schemes in its adaptation to different environments and bacterial hosts.

## Introduction

The IncF incompatibility group comprises a diverse set of conjugative plasmids frequently found in enterobacterial species like *E. coli* and *Salmonella*. This group was named after F: the factor found by Joshua Lederberg to be responsible for bacterial conjugation in *E. coli* K-12 (Lederberg and Tatum, 1946). The F factor was originally thought to be involved in some sort of para-sexual reproduction in *E. coli* (Makela et al., 1962), thus it was originally named the fertility factor, or F. Bacterial strains able to transmit genetic traits by conjugation were deemed fertile, or F^+^. It was also believed that fertility was different from R factors: self-transmissible episomes conferring antibiotic resistance to their hosts (Watanabe, 1967; Meynell et al., 1968a). Soon it was found that many R factors were sensitive to male-specific phages that infected F-bearing cells (Brinton et al., 1964; Caro and Schnös, 1966; Dennison, 1972). Serological testing revealed that many of these plasmids produced immunological cross-reactions (Orskov and Orskov, 1960; Ishibashi, 1967). Besides, it was observed that they were often unable to co-reside within the same recipient cell (Meynell et al., 1968b). Thus, it was concluded that F and some R plasmids constituted a distinct group, probably sharing a similar genetic structure, and a common ancestor (Meynell et al., 1968a). With the advent of DNA sequencing techniques, this idea was partially confirmed: IncF plasmids share a common set of genes involved in the genesis of the conjugative pilus. This is the reason behind their common phage sensitivity profile and serological cross-reactivity. Besides their common mating apparatus, F-like plasmids appear to be functionally diverse. For instance, they may encode different replication and partition systems (Ogura and Hiraga, 1983; Gerdes and Molin, 1986), and a wide diversity of cargo genes (Lanza et al., 2014; Johnson et al., 2016).

The F pilus is thus the common denominator of the IncF/MOB_F12_ group. F pili are distinct from other sex-related pili, such as the P, N, W, or X pili (4). The conjugation regions of these plasmids show similarity at the protein level to the VirB system of *Agrobacterium*, constituting prototypic Type IV secretion systems (T4SS) (Krause et al., 2000; Smillie et al., 2010; Chandran Darbari and Waksman, 2015). The F-pilus, however, is a more distant relative from VirB systems, albeit a true T4SS (Lawley et al., 2003). Unlike the short, rigid VirB-like pili, F pili are long and flexible, and able to retract upon contacting a recipient cell (Clarke et al., 2008). The genetic region involved in F conjugation is significantly longer and contains more genes than those of VirB-like pili forming plasmids (roughly 34 kb vs. 15 kb) (Kennedy et al., 1977; Frost et al., 1994; Lawley et al., 2003). One of its most conspicuous features is that all *tra* genes are transcribed from a single promoter (Helmuth and Achtman, 1975). The *tra* operon spans nearly 40 kb, making it, to the best of our knowledge, the longest transcript ever found in *E. coli*. Despite this simple operon arrangement, regulation of the transfer functions in IncF/MOB_F12_ plasmids is complicated. Expression of F conjugative functions is controlled by three transcriptional regulators: TraM, TraJ, and TraY (Frost and Koraimann, 2010; Arutyunov and Frost, 2013). From these three proteins, TraM and TraY play an additional role in relaxosome assembly (Wong et al., 2012; Lang et al., 2014). TraJ is the key activator of the Py promoter, responsible for the transcription of *tra* genes (Finnegan and Willetts, 1973; Frost and Koraimann, 2010). TraJ is regulated at the translational level by a small antisense RNA, FinP. FinP binds *traJ* mRNA, blocking its translation (Timmis et al., 1978; Arthur et al., 2003; Mark Glover et al., 2015). This process is assisted by the action of a key RNA chaperone, FinO (Ghetu et al., 2000). The *finOP* regulatory system constitutes the major controller of *tra* expression, and thus was named fertility inhibition system (Mark Glover et al., 2015). Besides this plasmid-encoded system, a relatively large number of host-encoded factors also modulate the expression of F transfer functions. In classical F-like plasmids, the key host factors regulating transfer expression are the transcriptional regulators ArcA, which co-activates the Py along with TraJ, and HNS, which acts as a silencing factor of the PY promoter. Besides these, other host factors like Lrp (leucine responsive regulatory protein), ArcB (anaerobic repressor of the arc modulon) and RNase E have been shown to modulate the expression of *tra* functions (Frost and Koraimann, 2010). The action of these host-encoded factors is often plasmid-specific, thus not all IncF/MOB_F12_ plasmids are linked to the host regulatory network in the same fashion. Paradoxically, the oddest case among naturally-isolated IncF plasmids is factor F itself. The F plasmid is a *finO*- mutant, produced by insertion of a IS3 insertion sequence (Yoshioka et al., 1987). Thus, it contains a non-functional fertility inhibition system, and exhibits conjugation frequencies two or three orders of magnitude above other naturally occurring IncF/MOB_F12_ plasmids like R1, R100, or pSLT (Frost and Koraimann, 2010).

These three plasmids (R1, R100, and pSLT) are considered “classical” IncF plasmids because they were extensively studied in the pre-genomic era. Plasmid R1 was transferred from its original host *Salmonella enterica* (serovar Paratyphi) to *E. coli*, conferring resistance to ampicillin, kanamycin, chloramphenicol and sulfonamides (Meynell and Datta, 1966). Plasmid R100 (also named NR1) was isolated from *Shigella flexneri* 2b, and encoded resistances to chloramphenicol, tetracycline and streptomycin (Nakaya et al., 1960). It was later found that R100 also provided the host cell with resistance to organomercury compounds (Womble and Rownd, 1988). pSLT was intensively studied because its role in the virulence of *Salmonella enterica* (serovar Typhimurium). Although the repertoire of classical F-like plasmids was reduced, it was observed that these plasmids presented significant differences in the regulation of the conjugative functions. With the advent of next-generation sequencing techniques, the genomes of hundreds of plasmids similar to classical IncF prototypes became available. Indeed, systematic studies of *E. coli* epidemic clones, like the widely distributed ST131, revealed an extraordinary prevalence of IncF plasmids in natural isolates (Lanza et al., 2014). This opens a number of questions regarding the conservation of IncF *tra* functions, and specially the regulatory circuits governing them. It is not known, for example, whether *finO*- plasmids like F itself are frequent among natural populations, or whether alternate regulatory schemes of the F conjugation machinery exist in nature. Using the relaxase as a phylogeny marker, we identified 256 IncF/MOB_F12_ plasmids in the NCBI plasmid database. By comparing the genomic structure of their conjugation regions, we identified five major groups displaying idiosyncratic genetic structures and alternative regulatory schemes. These five groups correspond to well-supported branches in the relaxase phylogenetic tree, indicating that these five groups represent radiations of an ancestral MOB_F_ conjugation system.

## Materials and methods

### Selection of the plasmid dataset

In order to identify MOB_F12_ plasmids, an initial search using a set of 26 known MOB_F12_ relaxases (Table S1) taken from previous studies (Garcillán-Barcia et al., 2009; Alvarado et al., 2012) was carried out. MOB_F12_ relaxases were defined as those having (D/E)NYY and D(L/F)TF amino acid motifs in the N-terminal relaxase domain of the protein (Garcillán-Barcia et al., 2009). These 26 relaxases were used as baits in protein BLAST searches of the NCBI plasmid database (6079 plasmids, 20th October 2015) using a threshold *e*-value of 1E-25. In this way, we retrieved a total of 256 plasmids containing relaxases that showed at least 40% sequence identity at the protein level to its closest database reference. This threshold was selected upon realizing that relaxases with low identity (down to 26%) were retrieved in an initial search. However, when we compared two of the most distant MOB_F12_ plasmids, for instance, F and pAsa5, they showed an ID of 49%. We therefore established as selection criteria that IncF/MOB_F12_ plasmids are those whose relaxase showed at least 40% ID with respect our homemade MOB_F12_ relaxase DB.

### Construction of protein profiles

To construct the presence/absence profile of the *tra* proteins of MOB_F12_ plasmids, Psi-blast searches (Altschul et al., 1997) were performed against a protein database constructed from the annotations of the 256 MOB_F12_ plasmids (Table S1) using the following conjugative proteins as queries: F plasmid proteins TraJ, TraA, TraL, TraE, TraK, TraB, TraP, TrbD, TrbG, TraV, TraR, TraC, TrbI, TraW, TraU, TrbC, TraN, TrbE, TraF, TrbA, ArtA, TraQ, TrbB, TrbJ, TrbF, TraH, TraG, TraS, TraT, TraD, TrbH, TraI (N-terminal 300 amino acids), and TraX as well as TraM, TraY, and FinO from plasmid R100. By default, hits below an *e*-value of 1E-3 were considered as positive hits. Selected protein hits were aligned using MUSCLE (Edgar, 2004). The resulting global alignments were used to reconstruct maximum-likelihood (ML) phylogenies using RAxML version 7.2.7 (Stamatakis, 2006). Twenty ML trees were executed using the JTTGAMMA model, and 100 bootstrap trees were inferred to obtain the confidence values for each node of the best ML tree.

### Co-occurrence matrix

To compute the co-occurrence matrix between *tra* genes, we used the presence/absence profile of all *tra* genes in our 256 plasmid dataset. Thus, for each *tra* gene, we defined a vector of 256 elements, with values 1 or 0. We then calculated the Hamming distance between each pair of vectors. Co-occurrence between two *tra* genes was expressed as the maximum possible distance (256) minus the Hamming distance between the pair of genes.

### Structural modeling

The 3D structures of TraJ_V_, EntFR, and SphTR were predicted by homology modeling using the Phyre2 server (Kelley and Sternberg, 2009) Images of the resulting 3D models were generated using Pymol (DeLano Scientific, Palo Alto, CA, USA).

## Results

### The MOB_F12_ phylogenetic tree includes plasmids from α and γ-proteobacteria

**Figure 1:**
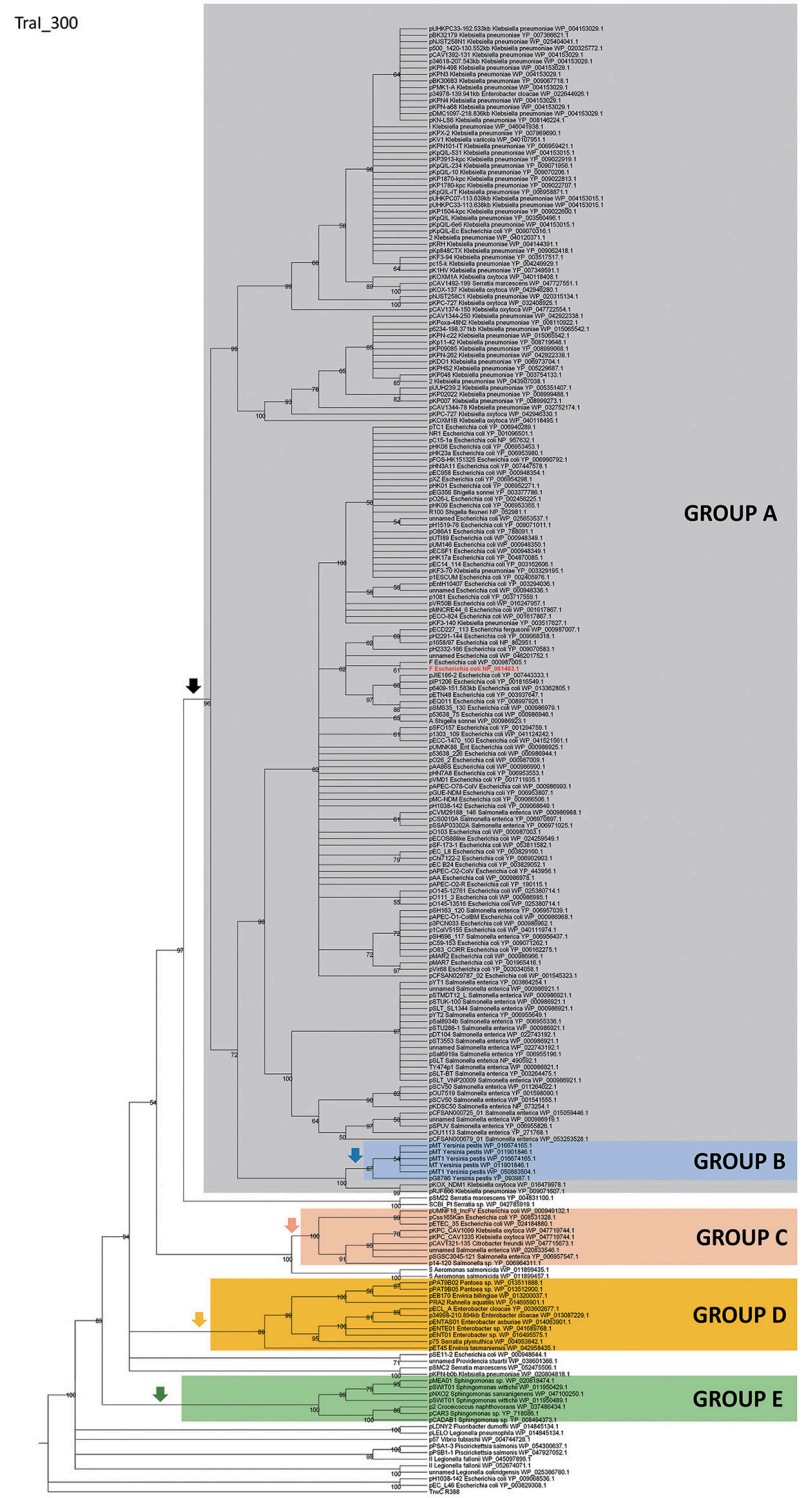
**Phylogenetic tree of MOB_F12_ relaxases**. The maximum-likelihood (ML) phylogenetic tree was built with the relaxase domain (N-terminal 300 residues) of 260 TraI_F homologs encoded by 255 plasmids present in our dataset (plasmid pCFSAN029787_01 relaxase was left out because it lacks the N-terminal relaxase domain). Bootstrap values are indicated at the corresponding nodes of the ML tree. The cut-off value for the condensed tree was chosen at bootstrap value = 50%. For each taxon, the plasmid name, the bacterial host, the GenBank protein accession number (excluding 13 non-annotated relaxases), and the GenBank plasmid accession numbers are indicated. The MOB_F12_ prototype (TraI_F) is highlighted in bold red letters. The MOB_F11_ relaxase TrwC of plasmid R388 (the first N-terminal 300 residues of GenBank Acc. No. FAA00039.1) was used as outgroup. Branches containing MOB_F11_ relaxases are drawn in gray. The MOB_F12_ cluster is indicated in the corresponding ancestral node. MOB_F12_ groups according to the organization and regulation of the conjugation system (A-E) are shadowed in different colors.

In order to identify IncF/MOB_F12_ plasmids present in the databases, we used the conjugative relaxase gene as the lowest common denominator. This property of the relaxase to serve as classification guide was shown in previous works, and is widely used for plasmid typing (Garcillán-Barcia et al., 2009; Alvarado et al., 2012). In order to identify MOB_F12_ relaxase-containing plasmids present in the NCBI plasmid database (6079 plasmids; October 20, 2015), we employed a set of 26 known MOB_F12_ relaxases as baits (Table S1), as indicated in Materials and Methods. We retrieved a total of 256 plasmids containing relaxases that showed at least 40% sequence identity at the amino acid level to their closest database reference (see M&M). The resulting plasmid list is shown in Table S1. To reconstruct the phylogeny of MOB_F12_ plasmids, we aligned the N-300 residues of the relaxase proteins as described in Materials and Methods and constructed a ML phylogenetic tree. We rooted the tree using the MOB_F11_ relaxase TrwC_R388 as outgroup. The resulting tree is shown in Figure 1. The MOB_F12_ phylogenetic tree includes plasmids isolated from α and γ-Proteobacteria, with 91% coming from species within *Enterobacteria*. The tree showed that 88% of the plasmids coming from enterobacterial species clustered in a monophyletic branch, well-supported by the bootstrap value (Figure 1, black vertical arrow). This branch included the “classical” IncF plasmids F, R1, R100, and pSLT. Enterobacterial plasmids not belonging to this branch included several from *Enterobacter*, which instead clustered in a second monophyletic branch (Figure 1, orange arrow). A third set of plasmids from *Escherichia, Salmonella*, and *Klebsiella* appeared in a third monophyletic branch (Figure 1, red arrow). As we will show later, these clusters contain plasmids harboring a typical MOB_F12_ conjugation region, but showing different regulatory systems. Plasmids from α-Proteobacteria appeared in an ancestral, monophyletic group (Figure 1, green arrow).

### Relaxases of the MOB_F12_ branch are associated to F-like conjugative systems

**Figure 2:**
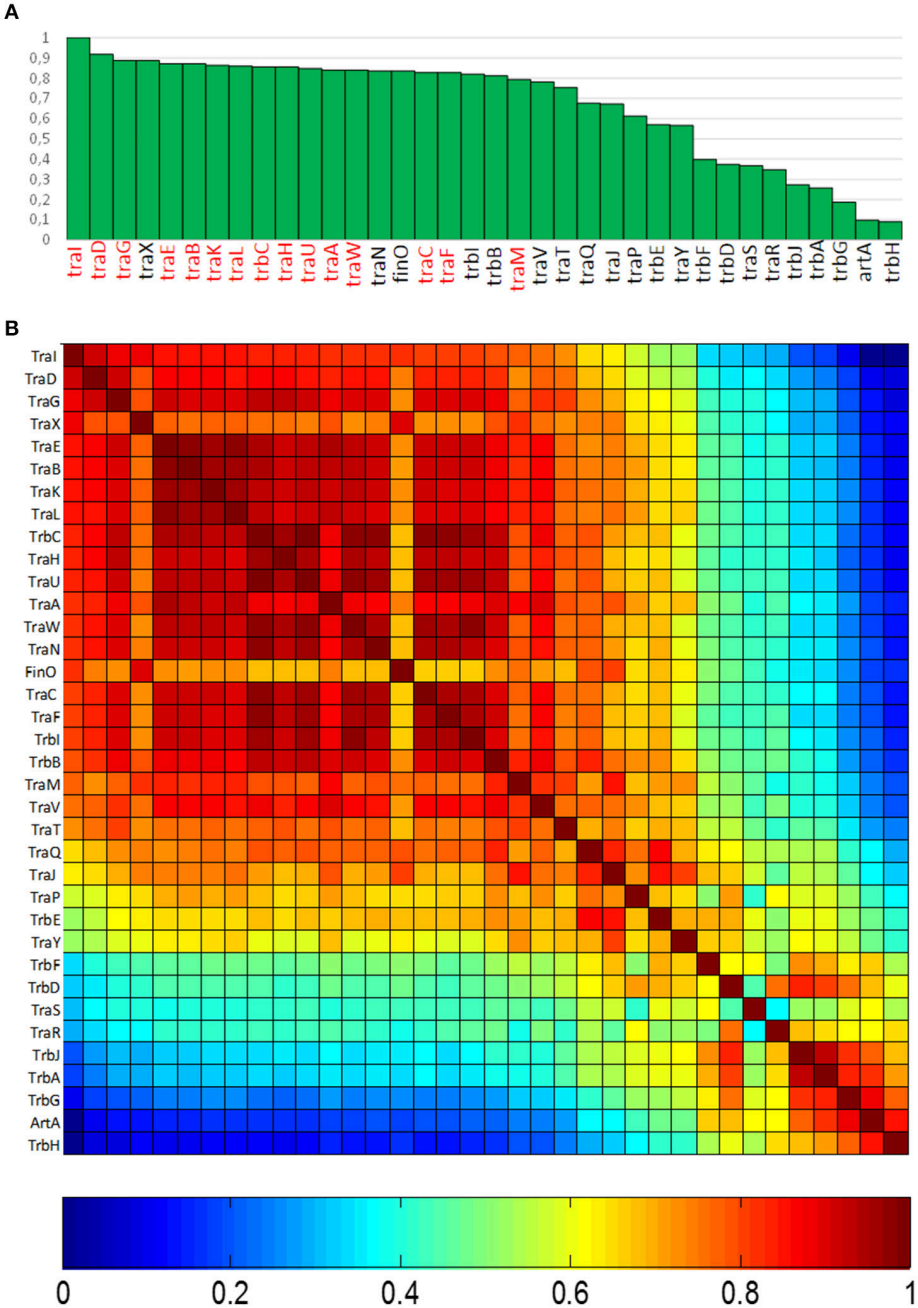
**Conservation of MPF_F_ conjugation genes in MOB_F12_ plasmids. (A)** The graph shows the percentage of plasmids (y axis) showing a gene homologous to each of the MPF_F_ genes indicated in the x axis, for the 256 plasmids that showed a MOB_F12_ relaxase. Genes were ordered according to their overall conservation. **(B)** Co-occurrence matrix of MPF_F_ genes in the 256 MOB_F12_ plasmids. The color matrix indicates the probability of co-occurrence (1, always appearing together in the same plasmid, 0 never appearing together in the same plasmid) for all MPF_F_ gene pairs.

In order to determine the degree of association of MOB_F12_ relaxases to the canonical F pilus, we determined the presence/absence of homologs of the F conjugation genes in our 256 plasmid dataset. For this purpose, we selected a total of 36 genes present in the conjugation region of plasmids F and R100, two well-studied IncF prototypes. As described in M&M, the presence of homologs of these 36 reference genes was determined by PSI-BLAST. Table S1 shows the accession numbers of each homolog identified for the 256 plasmids analyzed. Table S1 consists of a matrix in which rows (i) correspond to each MOB_F12_ plasmid, while columns (j) indicate each *tra* gene. Thus, reference numbers in the i,jth position of the matrix correspond to the homolog to protein j present in plasmid i. We transformed this table into a binary matrix, such that each i,jth position was 1 if there was a homolog detectable by PSI-Blast, and 0 otherwise. This allowed us to determine the level of overall conservation of F conjugation genes among the 256 plasmids. Results showed that a TraD-like protein (the coupling protein (T4CP) of F-like plasmids) could be detected in 91% of the plasmids (Figure 2A). This intimate phylogenetic association between the relaxase and the coupling protein was shown previously to be a hallmark of *mob* genes (Fernández-López, et al., 2006; Garcillán-Barcia et al., 2011). The analysis of other plasmid groups showed, however, that the association between MOB and MPF genes is less stringent. For example, MOB_F11_ relaxases are associated to N or W pili (variants of MPF_T_) (Fernández-López, et al., 2006; Garcillán-Barcia et al., 2011). As shown in Figure 2A, the presence of MPF_F_ genes could be detected in more than 80% of the MOB_F12_ plasmids in our dataset. Results also indicated that the conservation of MPF_*F*_ genes was not uniform. The most conserved MPF gene was *traG*, responsible for mating pair stabilization, which appeared in 88% of the plasmids. The least conserved gene was *trbH*, which could only be detected in 8% of the plasmids. Interestingly, genes that have been described as essential for F transfer appeared in more than 80% of the plasmids (Figure 2A, highlighted in red), while non-essential genes tend to appear at lower frequencies (Figure 2A, in black). An exception to this rule was *traX*, a gene that encodes an acetylase of pilin subunits. This gene was deemed non-essential for F plasmid conjugation (Maneewannakul et al., 1995), yet it was detected in 88% of the plasmids analyzed.

Since not all MPF_F_ genes showed the same degree of conservation, we wondered whether there were genes that showed preferential co-occurrence. To determine this, we built a co-occurrence matrix for the 36 MPF_F_ genes (Materials and Methods). Results (Figure 2B) showed that the highest co-occurrence values corresponded to the gene clusters *traEBKL, trbCI, and traHUWNCF*. These genes are essential components for the synthesis and function of F-pili, and thus their co-occurrence suggests the presence of functional F transfer systems. Interestingly, genes *trbA, trbG*, and *artA* also showed a high level of co-occurrence, despite their overall conservation is among the lowest overall (<25% identity). This indicates that this gene cluster is specific of a certain set of F-like plasmids. It was also noteworthy that regulatory genes (*traM, traY, traJ*, and *finO*) showed a lower degree of co-occurrence than structural genes, suggesting that alternative regulatory mechanisms could exist for MPF_F_ conjugation systems.

### Clustering of F-like (MOB_F12_) plasmids based on key regulatory genes of the transfer region

Since regulatory genes showed lower conservation than structural genes, we looked specifically at three key regulatory genes, namely *traM, traJ*, and *finO*. We did not include *traY* because, given its small size (around 225 bp), it is often not properly annotated. In order to distinguish between alternative regulatory schemes and major deletions that might have eliminated a substantial fraction of the transfer region, we also included a marker gene for the presence of the MPF apparatus. For this purpose, we used the essential ATPase *traC*. Using these genes as guidelines, we identified five major groups of F-like plasmids, which corresponded to major branches in the MOB_F12_ phylogenetic tree.

### GROUP A: classical F-like plasmids. prototype: plasmid R1

**Figure 3:**
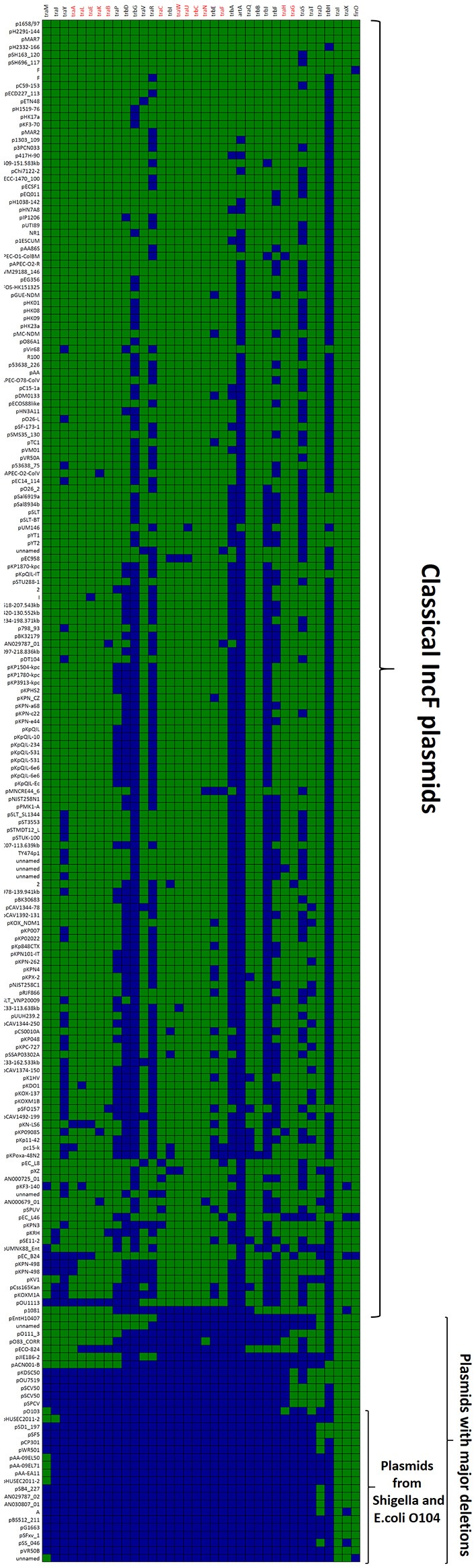
**Presence/Absence matrix of MPF_F_ conjugation genes in Group A plasmids**. Matrix columns correspond to the 36 MPF_F_ genes, while rows correspond to the 200 plasmids included in Group A. For each column/row combination, color green indicates the presence of the gene in the corresponding plasmid (PSI-Blast homolog identified with *E*-value below 10^−3^) while blue indicates its absence. Plasmids were ordered according to overall conservation.

The first and major MOB_F12_ group includes a total of 200 plasmids (78% of the total), residing in the genera *Escherichia, Salmonella*, and *Klebsiella*. In the MOB_F12_ tree of Figure 1, these relaxase genes are monophyletic. If we extrapolate from the relaxase to the whole TRA system, the tree structure implies that group A TRA_F_ system arose from a common ancestor that spread among these three bacterial genera. Plasmids of this group share a “classical” F plasmid conformation. Transfer genes are organized in a long, polycistronic operon, where gene synteny is preserved. Figure 3 shows the presence/absence table of *tra* genes in group A. It shows that we could detect the entire set of proteins deemed essential for F conjugation in a total of 150 plasmids. The essential genes, which were determined by transposon insertion analysis (Ippen-Ihler et al., 1972; Wu et al., 1987, 1988; Moore et al., 1990; Kathir and Ippen-Ihler, 1991; Maneewannakul et al., 1991, 1992; Maneewannakul and Ippen-Ihler, 1993), are shown in red in Figure 3. Remarkably, the regulatory components are also preserved among members of this group. TraM and TraJ appear in 100% of these plasmids. TraY homologs could be identified in 80% of the plasmids, but the real figure is probably higher, given the small size of the protein and lack of proper annotation. All MOB_F12_ group A plasmids were *finO*+, the only exception being plasmid F itself. Since the finO phenotype in the F plasmid is due to an IS3 insertion, it is thus highly likely that the “original” F plasmid was also repressed.

Within group A, a total of 50 plasmids lacked some essential component of the transfer machinery. Within this group, we identified a set of small plasmids (about 15 kb long) present in *Shigella* sp. and *E. coli* O104:H4 Shiga-toxin containing species (Figure 3, bottom). These plasmids seem to have suffered massive deletion of the TRA region, with only *traI, traX*, and *finO* genes remaining. This should result in a non-transmissible plasmid, since these plasmids neither contain the essential genes for pilus formation, nor the coupling protein TraD. In some cases, the relaxase itself appears truncated. The presence of plasmids with this particular structure among *Shigella* and Shiga-toxin containing *E. coli* is puzzling. The presumptive inability of these plasmids for horizontal mobilization would point out to the vertical propagation of a single deletion event in the ancestral line shared by *Shigella* sp. and *E. coli* O104:H4. However, members of this group do not form a monophyletic branch in the relaxase tree (Figure 1), which would suggest repeated but independent deletion events. Further research is needed to clarify the evolutionary history of these plasmids, the functional advantage of these deletions, if any, and their relationship to the pathogenesis of Shiga-toxin containing enterobacteria.

### GROUP B: MOB_F12_ plasmids from yersinia. prototype: pMT1

**Figure 4:**
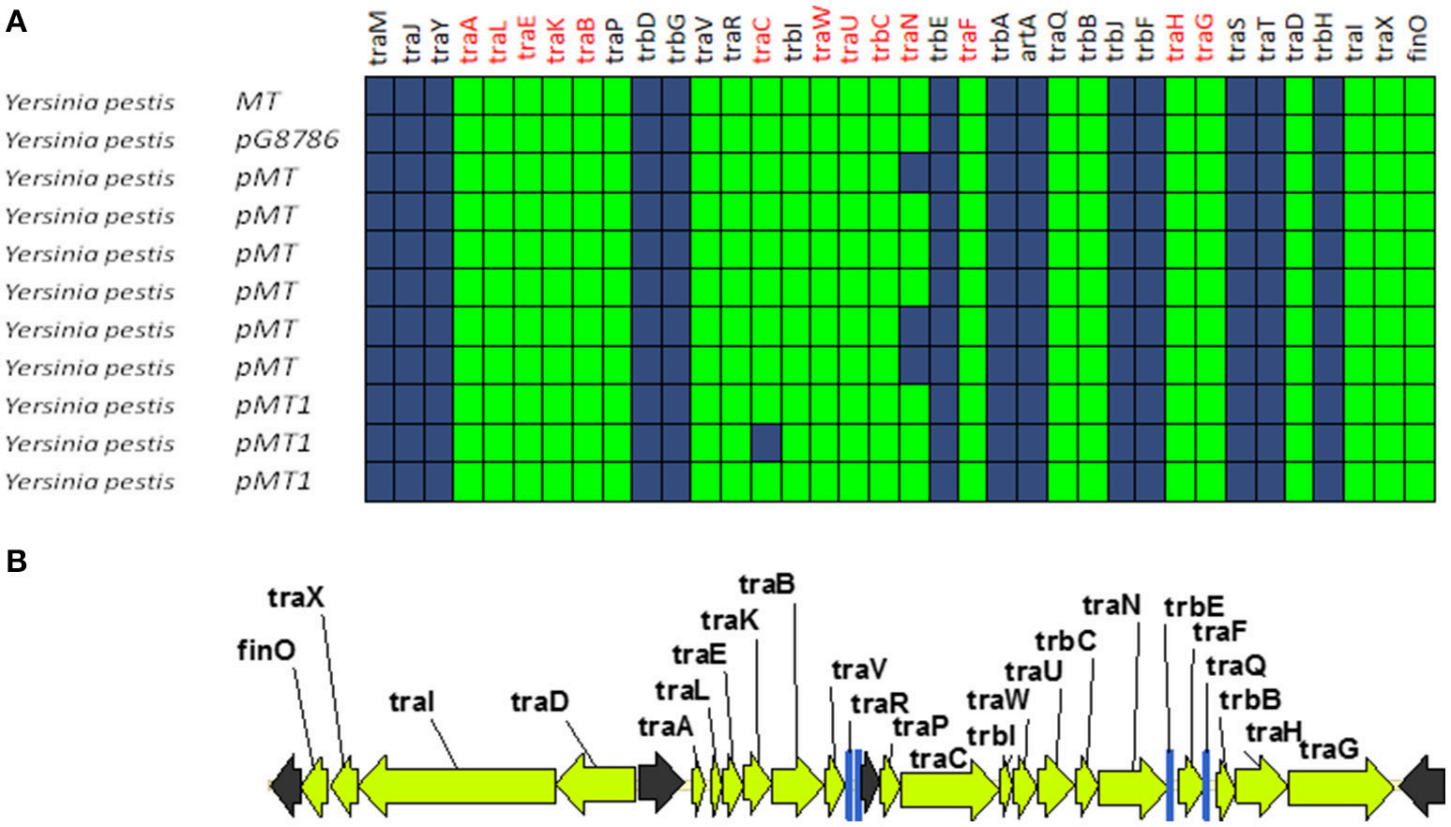
**Genetic structure and gene conservation in plasmids from Group B. (A)** Presence/Absence matrix of MPF_F_ genes for plasmids included in group B. Color green indicates the presence of the gene in the plasmid (PSI-Blast homolog identified with *E*-value below 10^−3^), while blue indicates its absence. **(B)** Genetic structure of plasmid pMT1, group B prototype. Yellow arrows and blue bars indicate ORFs corresponding to MPF_F_ genes conserved in other groups. Black arrows indicate ORFs for genes without detectable homology to other IncF-like plasmids.

A second group of F-like plasmids comprises a set of plasmids from *Yersinia pestis* (Figure 4A). Their relaxases appear as a monophyletic branch in the MOB_F12_ tree (Figure 1), showing an ancestral relationship to plasmids from group A (Figure 1). Structurally, they are characterized by a bipartite operon structure (Figure 4B), with genes involved in relaxosome formation (*traD, traI*) transcribed divergently from genes involved in conjugative pilus formation. Group B preserves all F essential genes and also *traP, traR, traR, trbI, traQ, trbB*, and *traX*. They all contain *finO*, yet none contain homologs of *traM, traJ*, or *traY*. Moreover, we could not find any putative transcriptional regulator in the vicinity of their conjugation regions, opening the questions of (a) how this mating system is regulated and (b) whether these plasmids are self-transmissible, given the lack of relaxase-accessory proteins or a recognizable origin of transfer. Outside the conjugation region, group B plasmids show extensive homology to plasmid pMT. pMT plasmids are a fundamental component of *Yersinia* pathogenesis, carrying essential virulence determinants for flea colonization (Hu et al., 1998). Besides, it is known that pMT plasmids from all three *Yersinia pestis* biovars (Antiqua, Medievalis, and Orientalis) are not self-transmissible and contain no transfer genes. Indeed, all *Yersinia* plasmids with a MOB_F12_ conjugation systems belong to isolates of *Yersinia pestis pestoides*, an atypical *Y. pestis* group, probably the closest to the ancestral lineage that gave rise to the pandemic biovars (Garcia et al., 2007). Incorporation of pMT to *Y. pestis* has been traditionally linked to horizontal gene transfer from other enterobacterial species (Hu et al., 1998; Lindler et al., 1998). According to the phylogeny shown in Figure 1, MOB_F12_ group B pMT plasmids stemmed from group A plasmids. Specifically, group B plasmids are monophyletic with two group A plasmids from *Klebsiella* sp. (pKOX_NDM1 and pRJF866) that contain the entire repertoire of essential F genes. However, given the lack of relaxase-accessory proteins (*traM* and *traY*), it is unclear whether group B plasmids are self-transmissible.

### GROUP C: MOB_F12_ plasmids related to IncFV. prototype: pUMNF18

**Figure 5:**
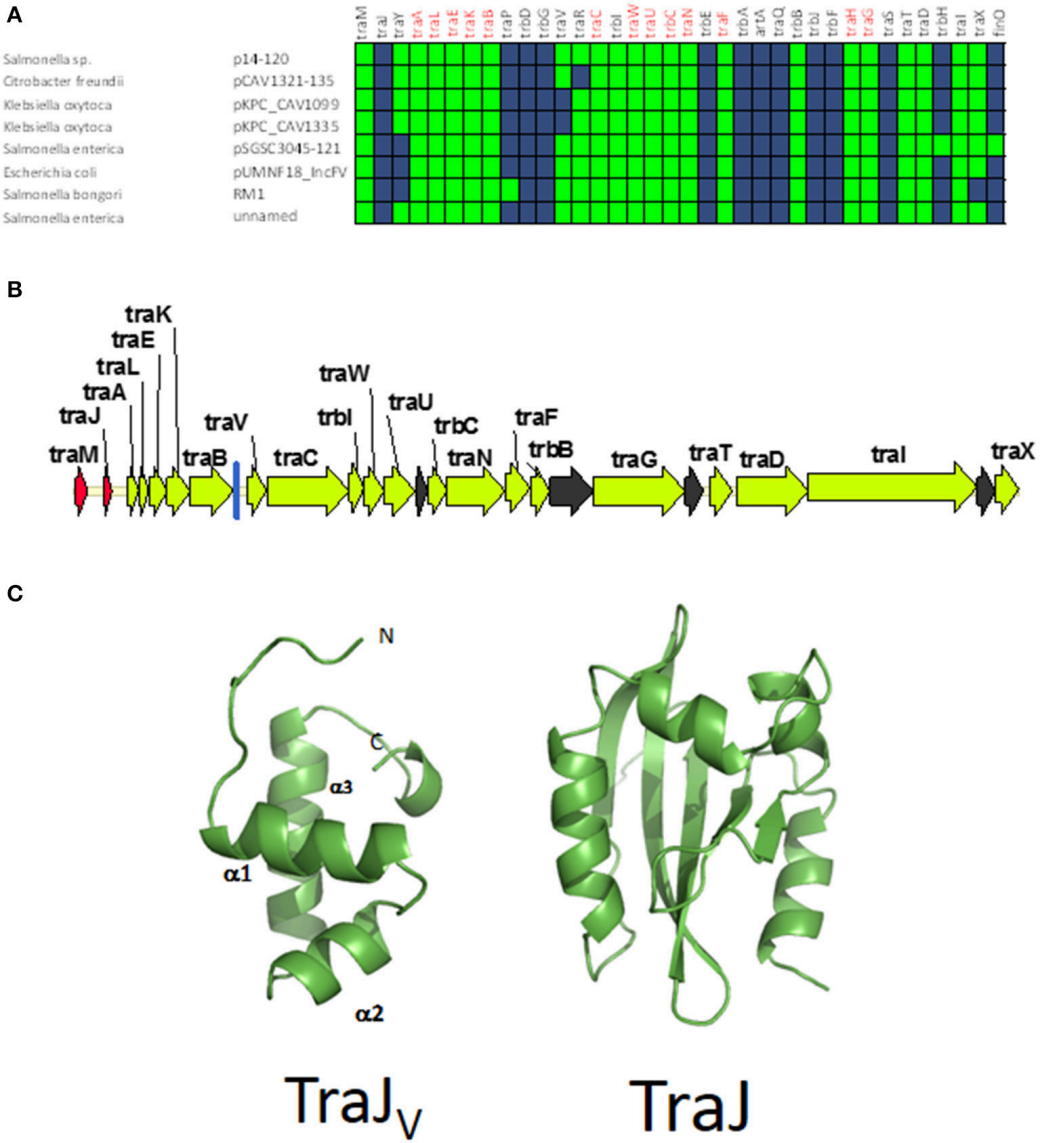
**Genetic structure and gene conservation in plasmids from Group C. (A)** Presence/Absence matrix of MPF_F_ genes for plasmids included in group C. Color green indicates the presence of the gene in the plasmid (PSI-Blast homolog identified with *E*-value below 10^−3^), while blue indicates its absence. **(B)** Genetic structure of plasmids plasmid pUMNF18, group C prototype. Yellow arrows and blue bars indicate ORFs corresponding to MPF_F_ genes conserved in other groups. Black arrows indicate ORFs for genes without detectable homology to other IncF-like plasmids. Red arrows indicate putative transcriptional regulators.**(C)** Structural prediction of the N-terminal domain of TraJ_V_ using the Phyre2 server (left) compared to the 3D structure of TraJ (right).

Although all plasmids from the MOB_F12_ group share a common mating apparatus (MPF_F_), only some IncF plasmids contain the same replication and partition machineries. Thus, some IncF plasmids are able to co-reside together in the same cell, while others are not. Classical incompatibility testing identified several IncF subgroups, which were numbered from I to VII (de la Cruz et al., 1979). One of these IncF subgroups, IncFV, stood out because of its particular regulatory scheme. In our analysis, we identified a set of plasmids that display a typical IncFV arrangement (Figure 5). Plasmids from group C form a monophyletic branch in the MOB_F12_ relaxase tree (Figure 1). They are characterized by a single promoter architecture. Conserved homologs include the same genes as in group A plasmids (Lu et al., 2002). The most conspicuous difference is that, although they encode a protein that is called TraJ, this protein actually shows no detectable homology to TraJ from Group A plasmids. Moreover, group C plasmids lack any recognizable homolog for FinO, yet experimental analysis showed that these plasmids are not de-repressed (Lu et al., 2002). To avoid confusion with the classical TraJ protein, hereafter we shall name this protein TraJ_V_.

According to structural analysis of TraJ_V_ with Phyre2, this protein is predicted to contain a DNA/RNA-binding domain (DBD) formed by a 3-helical bundle fold in the 70 N-terminal amino acids. This type of DBD is found in transcriptional regulators such as LuxR/UhpA family. Most LuxR-type regulators act as transcriptional activators. They contain an HTH domain in the C-terminal part of the protein and an effector binding domain in the N-terminal domain. However, in TraJ_V_ the HTH domain is located in the N-terminal half of the protein. A comparison between the predicted 3D structure for TraJ_V_ N-terminal domain and the solved structure for F plasmid TraJ N-terminal domain (pdb 4KQD) (Lu et al., 2014) showed no structural homology (Figure 5C). In fact, TraJ is predicted to be similar to the canonical LuxR protein, with an N-terminal effector binding domain and a C-terminal DBD. Thus, TraJ_V_ retains the DBD of TraJ, but the different position of this DBD and the differences in the rest of the protein suggest that TraJ_V_ could play a different role than TraJ in the control of MOB_F12_ group C plasmid conjugation.

### GROUP D: MOB_F12_ plasmids from enterobacter. prototype pENT01

**Figure 6:**
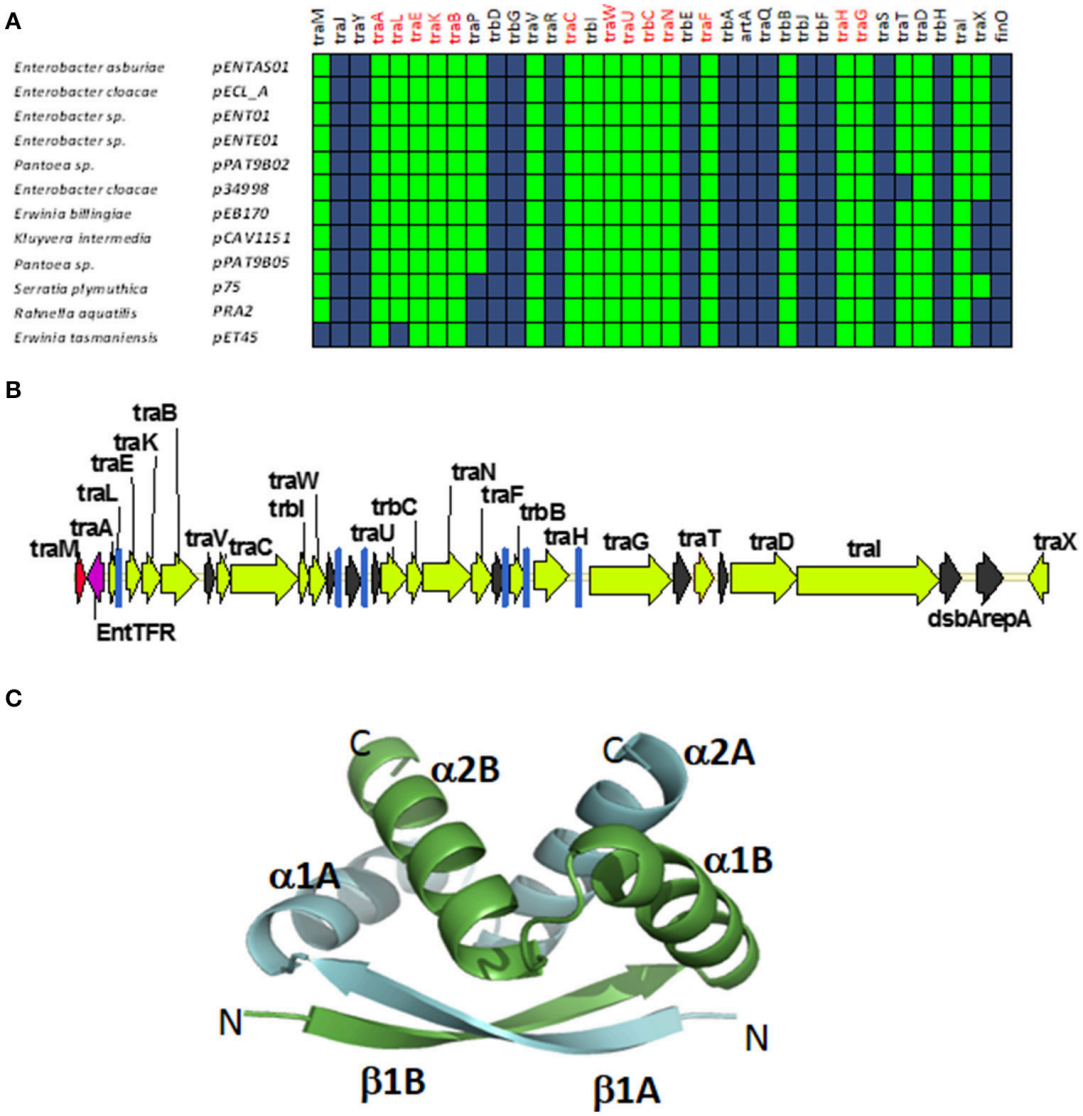
**Genetic structure and gene conservation in plasmids from Group D. (A)** Presence/Absence matrix of MPF_F_ genes for plasmids included in group D. Color green indicates the presence of the gene in the plasmid (PSI-Blast homolog identified with *E*-value below 10^−3^), while blue indicates its absence. **(B)** Genetic structure of plasmid pENT01, prototype of group D plasmids. Yellow arrows and blue bars indicate ORFs corresponding to MPF_F_ genes conserved in other groups. Black arrows indicate ORFs for genes without detectable homology among other IncF-like plasmids. Red arrows indicate putative transcriptional regulators. **(C)** Structural prediction of the putative regulator EntFR using Phyre2. The double RHH configuration is highlighted, with one RHH indicated in green (comprising the beta strand β1A and alpha-helices α1A and α2A), while the second RHH motif is indicated in blue (comprising the beta strand β1B and alpha-helices α1B and α2B).

A fourth group of F-like plasmids comprises members from another monophyletic branch in the MOB_F12_ tree. This branch includes plasmids mainly coming from *Enterobacter* and the closely-related genus *Pantoea*, but also contains plasmids from other enterobacteria like *Erwinia, Rahnella*, and *Kluyvera* (Figure 6A). These plasmids maintain the classical group A genetic organization: a single operon including all *tra* genes, except *traM* and *traJ* regulators, (Figure 6B). Genes that were deemed essential for F transfer are also conserved among plasmids of group D, along with *traV, trbI, traN*, and *trbB*. Remarkably, group D plasmids are FinO and TraJ negative. However, the locus occupied by *traJ* in Group A contains a DNA-binding protein that is conserved within the group. We named this putative regulator EntFR.

According to the Phyre2 prediction, EntFR is a ribbon-helix-helix (RHH) DBD (Figure 6C). Although this DBD is not as common as the helix-turn-helix (HTH) domain, it is frequently found in accessory proteins that bind to the origin of conjugative transfer (TrwA in plasmid R388, TraJ in plasmid RP4). In F plasmids TraM and TraY proteins present this fold. Structurally, EntFR is thus more similar to these proteins than to TraJ. EntFR contains two RHH domains, a feature that is also shared by TraY. Based on this, it is possible that EntFR acts as the functional homolog of TraY in group D plasmids. However, given that there are known RHH containing proteins able to act as transcriptional activators (Schreiter and Drennan, 2007), the possibility of EntFR being the functional homolog of TraJ cannot be ruled out.

### GROUP E: MOB_F12_ plasmids from sphingomonas. prototype pCAR3

**Figure 7:**
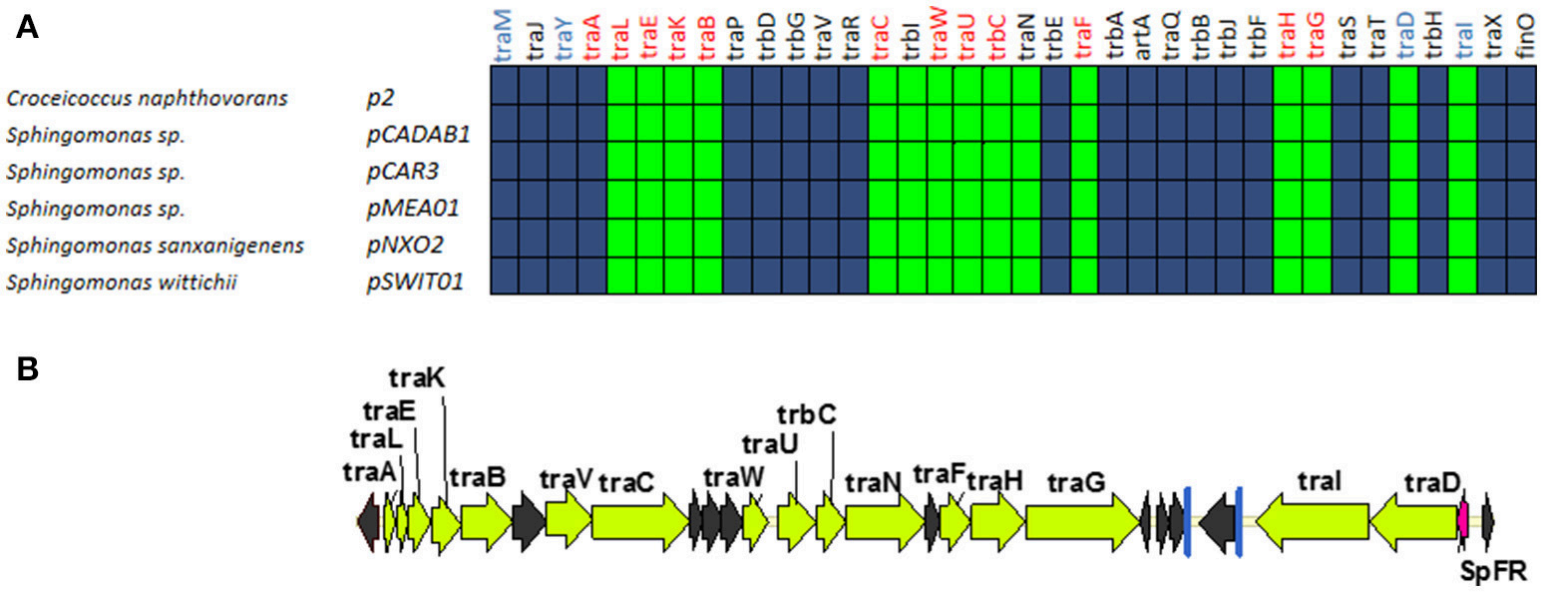
**Genetic structure and gene conservation in plasmids from Group E. (A)** Presence/Absence matrix of MPF_F_ genes for plasmids included in group E. Color green indicates the presence of the gene in the plasmid (PSI-Blast homolog identified with *E*-value below 10^−3^), while blue indicates its absence. **(B)** Genetic structure of pCAR3, prototype of plasmids included in Group E. Yellow arrows and blue bars indicate ORFs corresponding to MPF_F_ genes conserved in other groups. Black arrows indicate ORFs for genes without detectable homology among other IncF-like plasmids. Red arrows indicate putative transcriptional regulators.

A fifth group of F-like plasmids includes plasmids from the genus *Sphingomonas*, the relaxases of which form a monophyletic branch in the MOB_F12_ tree (Figure 1). These plasmids exhibit a conserved architecture that is different from the arrangement in the other MOB_F12_ groups (Figure 7). Instead of a long, single operon, *tra* genes from group E plasmids are split in two convergent operons. The first contains the genes responsible for the formation of the conjugative pilus (MPF_F_ genes), while the second includes the genes involved in relaxosome formation (Figure 7B). This architecture is reminiscent of that of conjugative plasmids with VirB-like mating apparatus, such as the MOB_F11_ (IncW and IncN) groups. However, although the architecture of group E is VirB-like, all the constituent genes are related to those of plasmid F, i.e., they belong to the MPF_F_ family. Genes deemed essential for F conjugation are preserved in group E plasmids, although *traA*, the gene coding for the conjugative pilin, is significantly different. Apart from these essential genes, group E plasmids also maintain clear homologs of *trbI* and *traN*. Regarding the regulatory components of the transfer machinery, group E plasmids lack homologs of TraM, TraJ, TraY, or FinO. The only putative regulator that can be identified by BLAST analysis is a small, conserved protein present immediately upstream *traD*. In plasmids with MPF_T_ (VirB-like) conjugation systems, this position is usually occupied by a protein coding for a ribbon helix-helix relaxase-accessory protein (e.g., TrwA in plasmid R388) (Moncalián and de la Cruz, 2004; Varsaki et al., 2009). Relaxase-accessory proteins participate in relaxosome assembly as well as in regulation of the expression of relaxosome components. In group E plasmids, this small protein can be thus considered as the hallmark of the group; it will be named SphTR (for Sphingomonas transfer regulator). Structural modeling of SphTR using Phyre2 showed that this protein belongs to to the ribbon helix helix (RHH) superfamily of DNA binding proteins. Thus, it is likely that SphTR fulfills the mobilization-accessory role in this group of plasmids.

Plasmids containing MOB_F11_ relaxases, like IncN and IncW plasmids, show an operon structure similar to group E plasmids. However, while in IncN and IncW plasmids it is possible to identify the regulators responsible for the independent control of the two operons (Fernandez-Lopez et al., 2014), we were unable to find any other putative DNA binding proteins in the vicinity of Group E transfer genes. Thus, it is unclear whether these genes are controlled by other plasmid/host regulators, by SphTR, or are not transcriptionally regulated. In any case, Group E plasmids constitute a valuable divergent evolutionary line of MOB_F12_ plasmids that, at least in genome organization and regulatory components, represents a bridge between MOB_F11_ and MOB_F12_.

### Other MOB_F12_ plasmids outside *Enterobacteriaceae*

A set of 14 plasmids remain unassigned in our group classification. They correspond to MOB_F12_ plasmids that were found outside the *Enterobacteriacea*e. Members of this group include plasmids from *Vibrio, Aeromonas, Legionella, Fluoribacter*, and *Piscirickettsia*. Although many of them contain an entire set of *tra* genes (like for example pLELO-like plasmids from *Legionella*), their genetic organization and putative regulators (indicated in Table S2) do not seem to be shared by the different plasmids (nor with Group E plasmids from *Sphingomonas*). It is entirely possible that this is due to under-representation of these genera in the nucleotide databases, compared to clinically-relevant enterobacteria. Anyhow, these plasmids serve to demonstrate that the IncF conjugation system is not restricted to the enterobacteria and that the MOB_F12_ plasmid clade can assimilate a number alternative of regulatory proteins.

### Comparison with other IncF typing systems: within group diversity

IncF plasmids are frequent carriers of antibiotic resistance genes and virulence factors, and a common finding in clinically relevant enterobacteria. Clinical microbiologists differentiate IncF plasmids using a sequence-typing system that takes advantage of the allelic diversity that IncF plasmids present in their replication regions (Villa et al., 2010). Analyzing replicon variants, Villa et al. were able to differentiate several IncF groups, according to their replicon sequence type (RST) (Villa et al., 2010). A comparison between replicon typing and the analysis of conjugation regions, revealed that plasmids with an assignable IncF RST belonged to Group A and Group B IncF plasmids, two groups that are monophyletic in the relaxase phylogenetic tree (Figure 1, black arrow). Group A plasmids, which include all classical IncF plasmids, present different RST profiles (Villa et al., 2010), indicating that within this broad group there is substantial sequence variation. Plasmids from groups C, D and E, however, cannot be assigned a typical RST profile, indicating that IncF plasmids in these groups are likely to exhibit different replication mechanisms.

## Discussion

The F plasmid was the first example of a conjugative plasmid found in bacteria (Lederberg and Tatum, 1946). IncF plasmids were also among the first plasmids known to provide antibiotic resistances (Meynell and Datta, 1966; Meynell et al., 1968b), colicins (Ozeki et al., 1962), and virulence determinants (Rotger and Casadesús, 1999). Because this historical relevance and their frequent association to clinically-relevant enterobacteria, F-like plasmids occupy a prominent place among bacterial plasmids. In order to analyze their conservation and diversity, we studied 256 plasmids that contained a MOB_F12_ relaxase. Analysis of these plasmids revealed that MOB_F12_ relaxases are associated exclusively with MPF_F_ conjugation systems. However, the MOB_F12_ conjugation systems analyzed presented a wider diversity than anticipated. Using the regulatory proteins as the most conspicuous indicators of this diversity, we could identify five major groups of IncF-like plasmids. As shown in Figure 1, these groups correspond to different branches of the relaxase phylogenetic tree. This indicates that these groups represent different radiations in the common branch of F-like plasmids. Interestingly, we also found a strong correlation between the MOB_F12_ phylogenetic groups and their bacterial hosts, suggesting that these groups might represent adaptations to different host genetic backgrounds. Group A was the most populated group, and included “classical” F-like plasmids like F, R1, pSLT and R100. Plasmids from this group are restricted to enterobacteria, with *E. coli, Klebsiella*, and *Salmonella* as the most frequent hosts. The overpopulation of this group compared to others, however, should not be taken as an indicator of particular evolutionary success. *E. coli, Klebsiella*, and *Salmonella* are clinically-relevant pathogens, much more represented in the genome databases than other species. Thus, the abundance of plasmids from group A could be just an indicator of sequencing bias. Plasmids from this group have been studied for decades, yet it yielded some surprising facts. First of all, it indicated how rare the F plasmid is. One of the motivations of this study was to determine whether de-repressed F-like plasmids were often found in clinical and environmental samples. Our analysis showed that de-repression by FinO inactivation is a property exclusive to the F-plasmid itself. Other genome alterations, particularly deletions, however, were far more common. At least 25% of the plasmids from group A lacked some of the genes deemed essential for F conjugation. This indicates that MOB_F12_ plasmids suffer frequent insertions and deletions, and that the presence of certain genes (such as the MOB_F12_ relaxase) cannot be taken as a guarantee that the plasmid is going to be self-transmissible.

Our results also revealed that some species are more prone to delete genes from the IncF conjugation region. The phylogenetic tree of Figure 1 indicates that these deletions can come from a single event, such as the monopyhletic plasmids from Group B in *Yersinia pestis*. In *Shigella* sp. and *E. coli* O104 even more radical deletions have occurred multiple times along the course of evolution. Importantly, all plasmids from these species showed plasmids with major deletions, indicating a strong selective pressure against MOB_F12_-conjugation genes.

Group A plasmids were the most common and the only ones to show a clear-cut fertility inhibition system (as judged from the presence of *traJ*/*finO* genes). Since group A is monophyletic, fertility inhibition was an innovation incorporated in some enterobacterial plasmid that then invaded *Escherichia, Salmonella*, and *Klebsiella* species. Although we cannot compare Group A abundance to groups outside the enterobacteria due to probable sequencing bias, it is interesting to note that there is another plasmid group which is exclusively found in enterobacteria, but much less populated. Group C plasmids, which include plasmids similar to classical IncFV plasmids, is restricted to the same species as Group A. This means that the sequencing bias between these two groups is less pronounced, yet Group A plasmids are much more abundant than Group C plasmids. Both plasmid groups share a common genetic structure, and their main difference is the presence of the fertility inhibition system. Thus, it is possible that the incorporation of the fertility inhibition system enhanced the ability of Group A plasmids to spread among enterobacterial species.

Groups D and E represent adaptations of the F conjugation machinery to other bacterial clades. Interestingly, the greater the phylogenetic distance between the hosts, the higher the level of divergence between IncF/MOB_F12_ plasmids. Thus, plasmids belonging to Group D are typically found in *Enterobacter* sp., a member of the *Enterobacteriaceae*, and their main difference with Group A plasmids is the presence of a different regulatory scheme, with EntFR likely playing the role of TraY. Meanwhile, Group E plasmids are present in Sphingomonas, an alpha-proteobacteria, and plasmids from this group present not only a different regulatory scheme, but also a different operon structure. Because their bipartite operon structure and the presence of a RHH protein in the same operon as the relaxase and the coupling protein, plasmids from this group resemble plasmids with MOBF_11_ relaxases, such as IncN and IncW plasmid groups (Fernández-López, et al., 2006). Judging from the relaxase phylogenetic tree, members of this group are also the closest phylogenetically to MOBF_11_ relaxases. This indicates that Group E plasmids represent an interesting intermediate link between VirB-like pilus containing plasmids and F-like pilus containing plasmids.

In summary, Groups A to E represent five alternate configurations for F-like plasmids. All these configurations present a shared protein core, which includes the 13 pilus genes deemed essential for plasmid F conjugation by the seminal work of Karin Ippen-Ihler (Ippen-Ihler et al., 1972; Maneewannakul et al., 1987, 1991, 1992, 1995; Wu et al., 1987, 1988; Moore et al., 1990; Kathir and Ippen-Ihler, 1991; Maneewannakul and Ippen-Ihler, 1993; Frost et al., 1994). Thus, while the mechanism of transfer is probably conserved, different regulatory mechanisms and operon structures exist in nature. It is also likely that other proteins not included in the *tra* operon, but known to play a role in conjugation like VirB1-like lytic transglycosylases show similar variation (Zahrl et al., 2005). This diversity has been found focusing exclusively in the conjugation region. However, it is known that plasmids belonging to group A present different replication and partition systems (Ogura and Hiraga, 1983; Gerdes and Molin, 1986; Villa et al., 2010). Thus, the exploration of the entire diversity of IncF plasmids requires analysis of other plasmid regions apart from the conjugation machinery. In particular, the diversity of replication strategies found among these plasmids is worthy of further analysis (Osborn et al., 2000; Villa et al., 2010). IncF plasmids are the most abundant plasmid type found in enterobacteria (de Toro et al., 2014). They are key to the generation and spread of clonal groups like *E. coli* ST-131 (Lanza et al., 2014; Johnson et al., 2016), and they are fundamental vehicles for the spread of antibiotic resistances (de Been et al., 2014). We hope that analysis using comparative genomics, like the one presented here, will help unraveling the causes behind the prevalence and evolutionary success of IncF plasmids.

## Author contributions

RF-L, MdT, GM, MPG-B, and FdC retrieved, analyzed the data, and wrote the paper.

## Funding

The work performed by the FdC group was supported by grants BFU2014-55534-C2-1-P and BFU2014-62190-EXP from the Spanish Ministry of Economy and Competitiveness and 612146/FP7-ICT-2013-10 from the European Seventh Framework Programme.

### Conflict of interest statement

The authors declare that the research was conducted in the absence of any commercial or financial relationships that could be construed as a potential conflict of interest.

## Supplementary material

Table S1 **(A)** List of MOB_F12_ relaxases used as bait in BLAST searches. **(B)** List of plasmids retrieved from the NCBI plasmid database. Columns D to AM indicate the NCBI reference number of the protein homolog for each of the F conjugation genes indicated in column headers, as explained in Materials and Methods.

Table S2 **Putative alternative regulators of MOBF12 transfer genes in groups C, D, E and plasmids not assigned to any group**.
